# Investigation of the Effects of Chain Extender on Material Properties of PLA/PCL and PLA/PEG Blends: Comparative Study between Polycaprolactone and Polyethylene Glycol

**DOI:** 10.3390/polym15092230

**Published:** 2023-05-08

**Authors:** Karabo Innocent Matumba, Mpho Phillip Motloung, Vincent Ojijo, Suprakas Sinha Ray, Emmanuel Rotimi Sadiku

**Affiliations:** 1 Centre for Nanostructures and Advanced Materials, DSI-CSIR Nanotechnology Innovation Centre, Council for Scientific and Industrial Research, Pretoria 0001, South Africa; kmatumba@csir.co.za (K.I.M.); mmotloung@csir.co.za (M.P.M.); vojijo@csir.co.za (V.O.); 2 Institute of NanoEngineering Research, Department of Chemical, Metallurgical and Materials Engineering (Polymer Division), Tshwane University of Technology, Pretoria 0001, South Africa; sadikur@tut.ac.za; 3 Department of Chemical Sciences, University of Johannesburg, Johannesburg 2028, South Africa

**Keywords:** PLA, PCL, PEG, blends, chain extender, morphology, mechanical properties

## Abstract

This study investigated the effect of the Joncryl concentration on the properties of polylactide/poly(ε-caprolactone) (PLA/PCL) and PLA/poly(ethylene glycol) (PEG) blends. The addition of Joncryl influenced the properties of both PLA-based blends. In the blend of PLA/PCL blends, the addition of Joncryl reduced the size of PCL droplets, which implies the compatibility of the two phases, while PLA/PEG blends showed a co-continuous type of morphology at 0.1% and 0.3 wt.% of Joncryl loading. The crystallinity of PCL and PEG was studied on both PLA/PCL and PLA/PEG blend systems. In both scenarios, the crystallinity of the blends decreased upon the addition of Joncryl. Thermal stabilities were shown to depend on the addition of Joncryl. The toughness increased when 0.5 wt.% of Joncryl was added to both systems. However, the stiffness of PLA/PCL decreased, while the stiffness of PLA/PEG increased with the increasing concentration of Joncryl. This study provides new insight into the effect of chain extenders on the compatibility of PLA-based blends.

## 1. Introduction

Managing solid waste that comprises polymeric materials from petrochemical resources such as thermoplastics and thermosets is a major global problem [1]. Polylactide (PLA) has received enormous attention and has been proven to be one of the potential alternatives to petroleum-based polymers, such as polyolefins [2]. PLA has good biodegradability, biocompatibility, and high mechanical properties [3,4]. PLA offers various applications in different sectors, such as packaging, biomedical, and structural [1,5,6,7,8]. However, PLA has inherent drawbacks, such as brittleness, slow crystallization rate, low melt strength, and difficulty processing at high temperatures, which restrict its wide range of applications [9,10]. To overcome these drawbacks, many approaches have been reported. This includes the incorporation of fillers, copolymerization, or blending with other polymers [11,12,13].

Blending is a more conventional and economical method for modifying polymer properties, which has been investigated by various authors [14,15]. Ductile polymers with a low glass transition temperature (Tg) can significantly improve the toughness of brittle polymers such as PLA by blending [15]. Biopolymers such as poly(ε-caprolactone) (PCL) and poly(ethylene glycol) (PEG) have been studied to improve the properties of PLA. PCL is a semicrystalline biodegradable thermoplastic with a low Tg of −60 °C, making it very flexible at ambient temperature [16,17]. However, PEG is another polymer that can be blended to improve PLA properties, thus broadening its potential applications. PEG is used primarily as a plasticizer to enhance the elasticity of PLA [18,19]. Although blending is considered a viable strategy to modify the properties of PLA, blend systems tend to be thermodynamically immiscible depending on the composition and processing conditions of the blend. This has mostly resulted in both matrices existing in separate phases caused by factors such as differences in the molecular weights and viscosity, which can affect the final properties of the system [20,21]. PLA/PCL and PLA/PEG blends are also thermodynamically immiscible.

Chain extenders have been developed to improve the compatibility of blend systems [22]. Chain extension is a chemical reaction of polymer molecules that uses the chain extender to expand the size of the molecules. The multifunctional chain extender, such as Joncryl, is one of the most effective chain extenders for polyesters, which can react with the carboxylic and hydroxyl end groups of polyesters [23,24]. During processing, chain extenders can maintain or even increase the molecular weight of polymers. This is because chain extenders reconnect polymer chains cleaved through the degradation reaction during processing [25]. Chain extenders can effectively improve polymer characteristics, such as the rheological, thermal, and mechanical behavior of polymers [26,27].

Several studies exist on the compatibilization of PLA/PCL and PLA/PEG blends using chain extenders. Doganci [28] used 1,4-phenylene diisocyanate (PDI) to improve the compatibility of PLA and PCL blends. A good increase in elongation-at-break was observed with a decrease in modulus. The morphology showed an improvement in the immiscibility of the two polymers, attributed to an improvement in mechanical properties. Bijarimi et al. [29] studied the mechanical properties of PLA/PEG blends and reported a decrease in strength, stiffness, and impact strength of PLA upon the addition of 2.5 to 10 wt.% PEG. Ghalia et al. [30] studied the effect of Joncryl on the properties of PLA-co-PEG. They reported an increase in tensile strength and elongation-at-break increased from 60 to 65 MPa and 10% to 16%, respectively. However, the modulus of PLA in PLA-co-PEG decreased from 1600 to 1580 MPa. The same co-workers, Ghalia et al. [30], further observed that 1.25 wt.% Joncryl in the PLA-co-PEG increased tensile strength, elongation-at-break, and modulus to 70 MPa, 17%, and 1680 MPa, respectively. 

This work investigates the effects of chain extension on the material properties of PLA/PCL and PLA/PEG blends. In this study, Joncryl was used as a multifunctional chain extender to act as a compatibilizer for PLA/PCL and PLA/PEG blends. Joncryl was used at different concentrations of 0.1, 0.3, and 0.5 wt.% to improve the thermal and mechanical properties, including the morphological characteristics of the resulting polymer blends. As a compatibilizer, the Joncryl epoxide group reacts with the carboxylic and hydroxyl end groups of PLA and PCL or PEG in the PLA/PCL and/or PLA/PEG blend systems. The blend composition of the PLA/PCL and PLA/PEG blends was fixed at 70/30 wt.%. The influence of the chain extender on the morphological characteristics and thermal and mechanical properties of the PLA-based blends was fairly investigated. PLA is expensive compared to other polymer commodities; hence, this work could be used to design other methods, such as foaming processes, to save material and costs. Therefore, both PLA/PCL and PLA/PEG blends were comparatively discussed to provide new insight on their material properties upon the inclusion of Joncryl as a chain extender.

## 2. Experiment and Methods

### 2.1. Materials

The PLA used in the study is commercial grade (PLA 3051D) and was obtained from NatureWorks, LLC, Plymouth, MN, USA. Celgreen PH-7 grade PCL was obtained from Daicel Chemical Industries Co., Ltd., Tokyo, Japan. PEG, with an average molecular weight of 20,000 g/mol, was obtained from Sigma-Aldrich, Johannesburg, South Africa. Joncryl ADR-4368 was obtained from BASF, Johannesburg, South Africa.

### 2.2. Preparation of Blends

Before processing, PLA was dried at 80 °C for approximately 24 h; PCL and PEG were kept at 30 °C for 24 h. The first step of this study was to optimize the best blend ratio in which that was obtained from PLA/PCL blends with different weight ratios. However, to choose the optimum blend, the weight ratios of the PLA/PCL blends studied were 80/20, 70/30, and 60/40. The PLA/PCL blends were processed by reactive blending using an extruder (Process 11, Thermo Scientific, Waltham, MA, USA) with a co-rotating twin-screw, length/diameter of 40 L/D, and a screw rotational speed of 150 rpm. The following temperature profile was used to prepare the blends, 70–100–150–160–170–170–165–165 °C from the feeding zone to the die. Therefore, the PLA/PCL blend ratio of 70/30 wt.% was chosen as the optimal blend. The chosen blend ratio is supported by the reported results on the morphological characteristics and mechanical properties as shown in Figures S1 and S2 (refer to the Supplementary Information). However, a PLA/PEG blend ratio of 70/30 *w*/*w* was intentionally chosen for good quality and provident comparison. The second step, which is the main purpose of this study, was to vary the different content of Joncryl in PLA-based blends (optimum blends). Therefore, the PLA-based blend ratios were maintained at 70/30 *w*/*w*, while the Joncryl content was varied at 0.1, 0.3, and 0.5 wt.% under the same conditions as the neat blends (Table 1). The content of Joncryl was chosen based on a previous study on PLA/PBSA blends compatibilized with Joncryl [24]. In their study, they studied gel permeation chromatography (GPC) to determine the molecular weight and dispersity of neat polymers containing chain extenders. The PLA-based blends obtained were compression-molded at a temperature of 190 °C and a pressure of 1 MPa for 6 min, using a Carver compression molder (Carver, model 973214A). After compression-molding, the samples were ready for characterization.

### 2.3. Characterization

Morphological analysis was performed using scanning electron microscopy (SEM) (JSM-7500, JEOL, Tokyo, Japan) at an acceleration voltage of 3 kV. Before analysis, the samples were fully dipped into the liquid nitrogen and then freeze-fractured. The freeze-fractured samples were carbon-coated to reduce charging.

Thermal stability studies were performed using thermogravimetric analysis (TGA) (TGA5500, TA Instruments, New Castle, DE, USA). Samples were weighed between 9 and 10 mg and then heated from 35 to 900 °C at a rate of 10 °C/min. The samples were heated using steel pans under an air atmosphere.

A differential scanning calorimeter (DSC) (DSC 8500, PerkinElmer, Waltham, MA, USA) was used to study the thermal transitions of the samples using a sample mass between 5 and 6 mg with a temperature ranging from −65 to 200 °C at a rate of 10 °C/min under a nitrogen atmosphere with a flow rate of 20 mL/min. The second heating and cooling scans were then analyzed to study the melting and crystallization parameters, respectively. The degree of crystallinity (X_c_) was calculated using Equation (1):

(1) $$X_{c}=\frac{{∆H}_{m}}{\omega \times {∆H}_{o}}\times 100\%$$

where ∆H_m_ is the specific melting enthalpy and ∆H_o_ is the melting enthalpy of 100% crystalline neat polymer, and where the melting enthalpy of 100% PLA is 93.7 J/g [31], for PCL is 136 J/g [32], and for PEG is 197 J/g [5]. Therefore, ω is the weight fraction of the PLA, PCL, and/or PEG phase in PLA/PCL and/or PLA/PEG blends.

X-ray diffraction (XRD) studies of compression-molded samples were performed using an X’Pert PRO diffractometer (PANalytical, EA Almelo, The Netherlands) that produces Cu Kα radiation (λ = 1.54 Å). The diffractometer was operated at 45 kV and 40 mA, and XRD patterns were recorded in the 2θ range of 5–90 degrees.

Thermomechanical properties of the compression-molded samples were studied using a dynamic mechanical analyzer (DMA) (DMA 8000, PerkinElmer, Waltham, MA, USA) under the dual-cantilever bending mode in the temperature ranging from −100 to 150 °C at a heating rate of 2 °C/min. Analyses were performed at a frequency of 1 Hz with a strain amplitude of 0.02%.

Tensile measurements of compression-molded samples were performed using an Instron 5966 tester (Instron Engineering Corporation, Norwood, MA, USA) with a load cell of 10 kN. The tests were carried out in tension mode at a single strain rate of 5 mm/min at room temperature. Dumbbell-shaped samples with dimensions 25 mm × 3.1 mm × 3.3 mm were used in the test, reporting the results of at least an average of six individual tests per sample.

## 3. Results and Discussion

### 3.1. Phase Morphology

**Figure 1:**
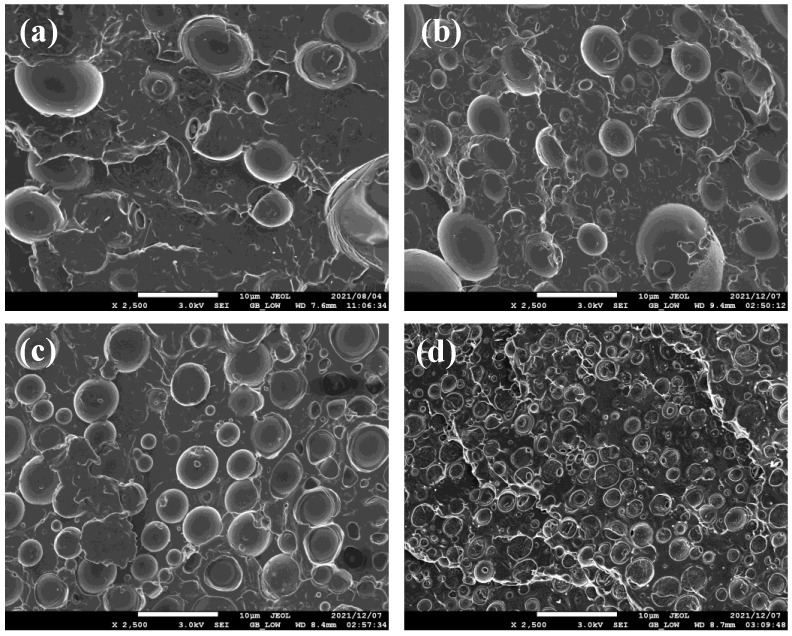
SEM images of the cryogenically fractured surface morphologies of PLA/PCL blends with varying content of Joncryl: (**a**) PLA/PCL 70/30, (**b**) PLA/PCL JC0.1, (**c**) PLA/PCL JC0.3, and (**d**) PLA/PCL JC0.5.

Figure 1 shows the evolution of the cryogenically fractured surface morphologies of PLA/PCL blends containing Joncryl at varying concentrations. Figure 1a reveals a matrix-dispersed morphology, where PCL droplets are dispersed in a continuous PLA matrix. This indicated the incompatibility between the PLA and PCL matrices. The morphology did not show any significant change after the addition of 0.1 wt.% Joncryl (Figure 1b). However, in Figure 1c,d, it was observed that when the Joncryl content increased by 0.3 and 0.5 wt.%, the size of the PCL droplets decreased, and they were homogeneously distributed within a continuous PLA phase. A decrease in the droplet size of the dispersed phase indicates compatibility of the components of the blend components [7,20]. From these observations, it is evident that 0.5 wt.% Joncryl had a compatibilizing effect. Similar results were reported by Dong et al. [33]: the addition of 1 wt.% Joncryl reduced PBAT droplets in the PLA/PBAT (80/20) blend, indicating a reasonable degree of compatibility between PLA and PBAT.

**Figure 2:**
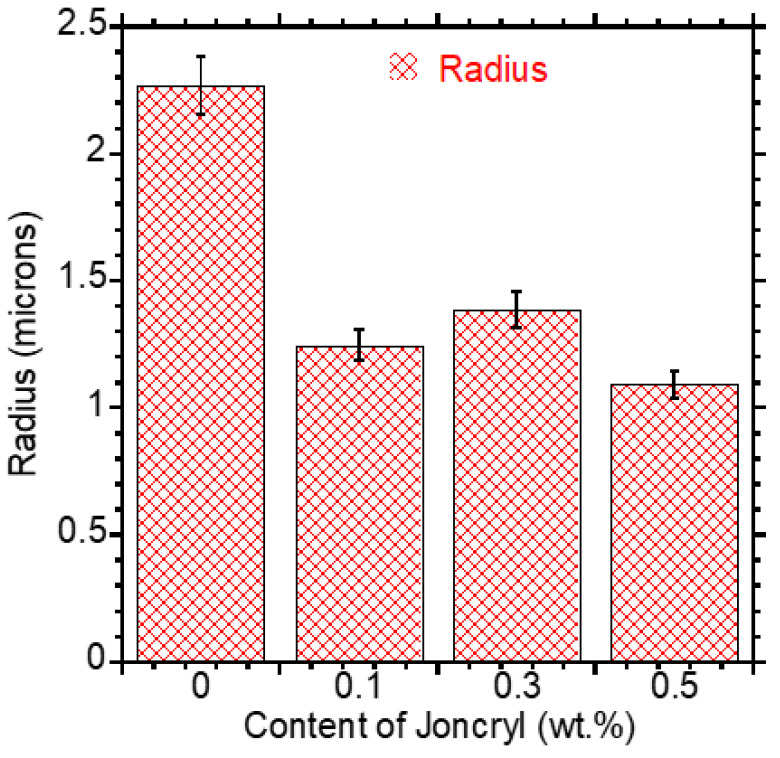
Plot showing particle size of PCL phase with varying Joncryl content in the PLA/PCL-based blend.

The droplet sizes were determined using ImageJ 1.53k software. Two images per sample were analyzed to determine the radius of PCL droplets. Figure 2 shows the effect of Joncryl content on the size of the dispersed phase. The average radius of PCL droplets in the PLA/PCL blend was 2.27 μm, which decreased with the incorporation of the chain extender. The smallest droplet size was noticed with the addition of 0.5 wt.% Joncryl, which showed a decrease to 1.09 μm. The size of the droplet can be explained by the viscosity ratio between a continuous PLA phase and a dispersed PCL phase (ղPLA/ղPCL). A reduction in the droplet size of the dispersion phase is substantially affected by the viscosity ratio and is usually observed when the viscosity ratio ≈ 1, in this case, that is, when ղPCL ≈ ղPLA [15,20]. According to Grace [34], the drop deformation for drop breakup occurs in the viscosity range from 0.1 to 1. However, the reduced shear rate required for breakup becomes smaller in irrotational (extensional) shear as the viscosity ratio moves away from 1 in either direction, compared to rotational shear. Therefore, when the shear rates are equal, irrotational shear tends to produce more effective breakup and dispersion than rotational shear, even at low viscosity ratios. At very low or high viscosity ratios, a high interfacial tension makes it several hundred times more difficult for droplets to break by uniform rotational shear. During blending, the dispersed phase forms threads, which break down into smaller droplets. This is then followed by the coalescing of small droplets to form large droplets. However, the coalescing is suppressed upon the inclusion of Joncryl, hence the formation of small droplets. In this case, 0.5 wt.% Joncryl effectively reduced the droplet size of PCL.

**Figure 3:**
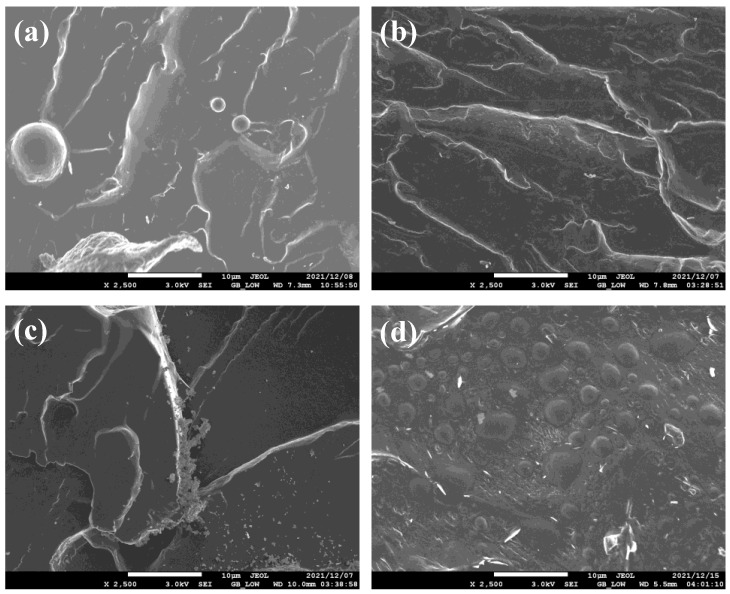
SEM images of the cryogenically fractured surface morphologies of PLA/PEG blends with varying content of Joncryl: (**a**) PLA/PEG 70/30, (**b**) PLA/PEG JC0.1, (**c**) PLA/PEG JC0.3, and (**d**) PLA/PEG JC0.5.

Figure 3 shows the SEM images of the cryogenically fractured morphologies of the PLA/PEG-based blends. The PLA/PEG blend (Figure 3a) showed few PEG droplets dispersed in a continuous PLA matrix. However, with the addition of Joncryl at 0.1 and 0.3 wt.%, it was difficult to differentiate between PLA and PEG since both appeared as co-continuous phases. This suggests compatibility between the blend matrices, as observed elsewhere [35]. At 0.5 wt.% Joncryl, a transformation to the development of matrix-dispersed morphology could be noticed. Therefore, a rougher surface was observed, as shown in Figure 3d. Ghalia et al. [30] reported a similar observation after the addition of a 1.25% chain extender in PLA-co-PEG. Therefore, the rough surface is due to the distinguished crystallite areas and amorphous phase of the PLA in the blend [36].

### 3.2. X-ray Diffraction Analysis

**Figure 4:**
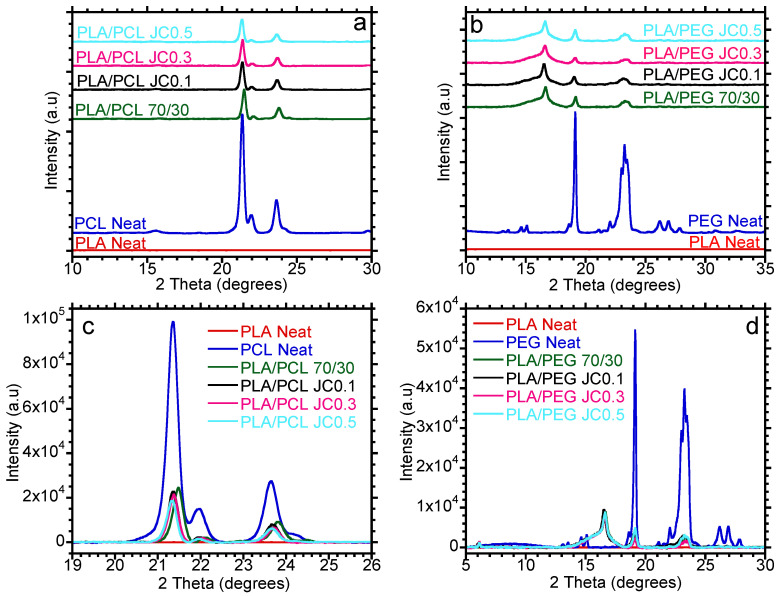
XRD of (**a**,**c**) PLA/PCL and (**b**,**d**) PLA/PEG blends with varying content of Joncryl at 0.1, 0.3, and 0.5 wt.%.

Generally, the material properties of polymeric materials depend on the crystallinity of the polymer. Crystalline polymer materials show better thermal characteristics, such as high heat distortion temperature and load-bearing properties. In this case, the influences of PCL and PEG on the PLA’s crystalline structure were evaluated using the WAXD. The crystalline structures of the prepared blends are depicted in Figure 4. PLA is amorphous in nature and does not show any crystalline peak. However, PCL and PEG are semicrystalline materials; therefore, they show diffraction peaks, as observed in Figure 4a,b. PCL is characterized by strong diffraction (2theta) peaks at 21.4° and 23.7°, which are associated with the (110) and (200) crystalline planes [37], respectively.

On the other hand, PEG shows strong diffraction peaks at 19.1° and 23.2°, which correspond to the crystalline planes of (120) and (032) [38], respectively. The blends of PLA/PCL and PLA/PEG show diffraction peaks related to either PCL and/or PEG. The PCL diffraction peaks could be observed in the neat PLA/PCL blend, although the intensity of the peaks decreased. The addition of Joncryl did not show any significant effect on the crystallinity of PCL in the blend. However, the intensity of the PCL crystalline peak in the blend slightly decreased with the addition of 0.5 wt.% Joncryl, insinuating a slight decrease in the crystallinity of PCL in the blend. On the other hand, the intensities of the PEG crystalline peaks at 19.1° and 23.2° decreased in the PLA/PEG blend. Therefore, the nucleating effect of PEG on PLA could be observed. The PLA/PEG blend showed the appearance of a crystalline peak at 2θ = 16.6°, which is related to PLA and corresponds to the (110) crystal plane (110) [5]. However, similar behavior could be observed with the addition of Joncryl. Athanasoulia et al. [35] reported similar results, in which they discovered an appearance of a crystalline peak at 16.6° with the addition of 30 wt.% PEG to PLA. This could represent platelets of different dimensions or α’-crystalline phase with lower packing caused by incomplete crystallization. However, increasing the concentration of Joncryl led to the deterioration of the crystalline structure of PEG in the PLA/PEG blend. Thus, it can be observed that higher concentrations of Joncryl impede the crystallization of PCL and PEG in the blend. A decrease in crystallinity could imply an increase in the toughness of the material. DSC investigations were carried out to study the crystallinity of the blends, and the results are discussed in the following section.

### 3.3. Thermal Properties

**Figure 5:**
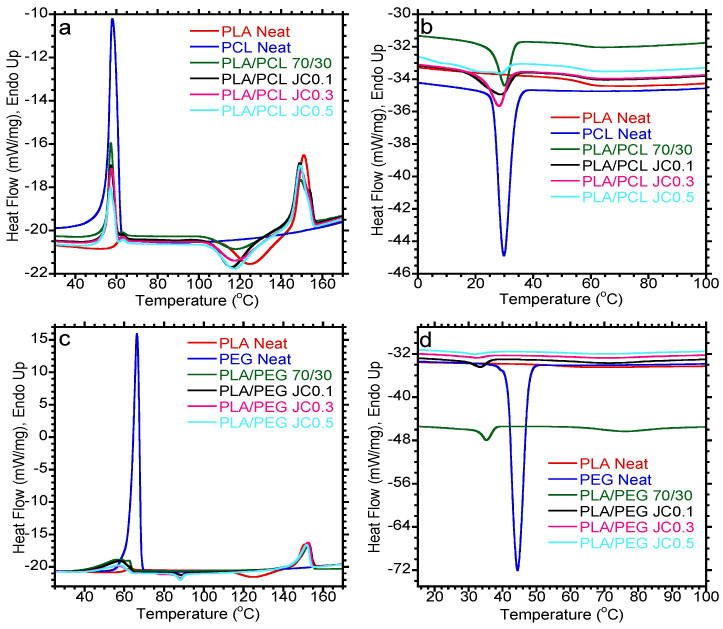
DSC (**a**,**c**) second heating and (**b**,**d**) cooling curves of PLA-based blends with varying content of Joncryl at 0.1, 0.3, and 0.5 wt.%.

Figure 5 shows the DSC heating and cooling curves for PLA/PCL and PLA/PEG-based blends with varying Joncryl content. The DSC data for melting, crystallizations, and degree of crystallinities are summarized in Table 2 and Table 3. The heating curves for PLA/PCL and PLA/PEG blends are shown in Figure 5a,c. Neat PLA is characterized by glass transition temperature (Tg) at 60 °C, a cold crystallization temperature (Tcc) at 125.9 °C, and melting temperature (Tm) at 151.0 °C. PCL and PEG show a melting temperature (Tm) of 57.8 and 66.1 °C, respectively. Blending PLA with PCL and/or PEG did not have a significant influence on the Tm of PLA. Additionally, the addition of Joncryl in both blend systems did not show any difference. However, the Tcc of PLA was significantly reduced upon blending. When PCL was introduced into PLA, Tcc decreased from 125.9 (neat PLA) to 119.1 °C, while the addition of PEG showed a dramatic decrease to 88.9 °C. The reduction in Tcc of PLA is attributed to the nucleating effect of both PCL and PEG. PCL and PEG improve the chain mobility of PLA, which increases the Tcc rate of PLA, allowing it to occur at lower temperatures. The addition of Joncryl to either of the systems did not influence the Tcc of PLA. Dong et al. [33] added Joncryl to the PLA/PBAT blend and discovered a decrease in Tcc of PLA. The Tcc of PLA decreased due to the heterogenous nucleation effect of the compatibilized PBAT domains on the PLA. The Tcc of PLA in the PLA/PEG blend did not occur. However, the Tm of PLA was still observed, implying that there is crystallization. In that case, normal crystallization was expected to occur during cooling, but that was not the case. This could be due to the slow crystallization rate of PLA.

The DSC cooling curves are shown in Figure 5b,d. PLA has a slow crystallization rate; hence, it did not show any crystallization temperature (Tc) during cooling. However, only the Tc of PCL and PEG could be observed during cooling. The neat PCL showed Tc at 30.1 °C, while PEG has Tc at 44.1 °C. The Tc of PCL was not significantly affected upon blending with PLA and when Joncryl was introduced. On the other hand, the Tc of PEG decreased to lower temperatures in the blend and further decreased upon the inclusion of Joncryl. This indicated a delay in the crystallization of PEG in the blend attributed to PLA inhibiting the rearrangement of the PEG chains to form the crystalline structure in the blend. The degree of crystallinity of PLA was determined and the results are summarized in Table 2 and Table 3. It can be observed that PCL and PEG influenced the crystallinity of PLA. Blending with PCL and PEG increased the crystallinity of PLA as noticed. It can be inferred that the addition of a low PCL content is sufficient to activate the crystallization of PLA. Todo et al. [39] also reported on the activation of PLA crystallinity after blending with PCL. However, in the case of the PLA/PEG blend, Shin et al. [40] also observed an increase in PLA crystallinity with the addition of PEG, suggesting that PEG imparted effective plasticizing properties to PLA in the blend. PEG increases the chain mobility of PLA, which increases the crystallization of the blend [41]. However, the crystallinity of PLA within the PLA/PCL blend increased with the addition of Joncryl, whereas a slight decrease in the crystallinity of PLA within the PLA/PEG blend was observed upon the addition of Joncryl. Ojijo et al. [42] observed similar results in which PLA crystallinity in the PLA/PBSA (70/30) blend increased with the addition of triphenyl phosphite (TPP) as a chain extender. This is attributed to the compatibility of PLA and PBSA, which suggests that the two polymers were partially miscible [42]. In conclusion, both PCL and PEG had similar effects on the thermal properties of PLA as noticed, albeit trends were observed in the respective degree of crystallinities.

### 3.4. Thermal Stability

**Figure 6:**
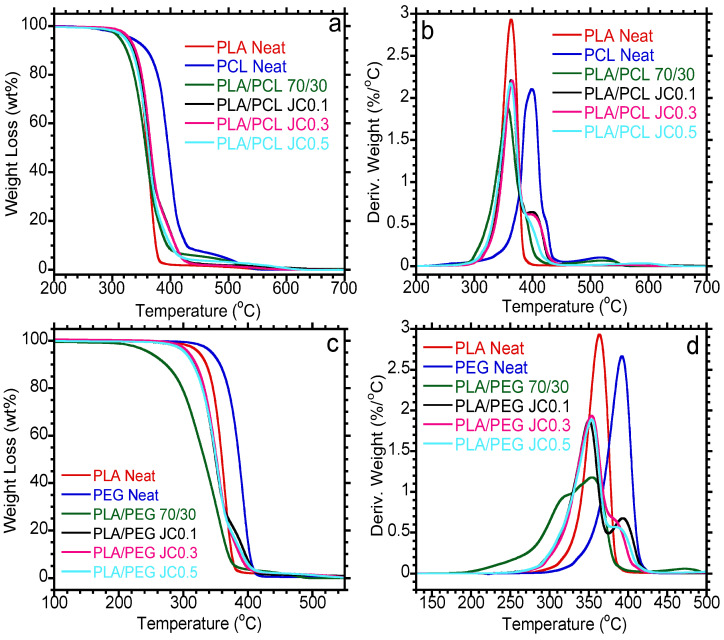
TGA (**a**,**c**) and dTGA (**b**,**d**) curves of PLA-based blend with varying content of Joncryl.

Figure 6 shows the TGA and dTGA curves of PLA/PCL and PLA/PEG-based blends containing Joncryl. The values of the maximum degradation temperatures (Tmax) of PLA, PEG, and PCL are summarized in Table 4. The neat polymers show single-step degradation, with Tmax observed at 362.2, 390.2, and 402.1 °C corresponding to percentage degradation at 39.9, 33.7, and 42.2% for PLA, PEG, and PCL, respectively. PLA has low thermal stability compared to both PEG and PCL. PLA/PEG and PLA/PCL blends, with and without Joncryl, are characterized by two degradation steps that correspond to the respective contents of each polymer within the blends. PLA has low thermal stability, and blending with either PEG or PCL is expected to increase its thermal stability. However, blending with PCL did not significantly influence the Tmax of PLA. In contrast, the inclusion of Joncryl showed a slight increase in the thermal stability of PLA, as observed in Table 4. However, the inclusion of PEG showed a reduction in Tmax of PLA, with a further decrease after the inclusion of 0.1 wt.% Joncryl. The decrease in the thermal stability of PLA in the PLA/PEG blend could be due to the presence of PEG in the blend [30]. An efficient plasticizer infiltrates the molecular chain, which in turn reduces the Tg of the amorphous region. Therefore, this increases the flexibility of the molecular chain, thus decreasing the thermal stability of PLA [40]. In addition, the addition of PEG makes PLA more sensitive to thermal stability in higher temperature ranges [5]. However, a further increase in the Joncryl content increased the thermal stability of PLA. In comparison of the two blend systems, it is apparent that the thermal stabilities of both PEG and PCL were influenced by blending with PLA. For PCL-containing blends, a reduction in Tmax from 402.1 to 377.4 °C corresponding to percentage degradation at 28.0% was noticed, while the Tmax for PEG decreased from 390.2 to 304.9 °C corresponding to percentage degradation at 77.0%. However, incorporation of Joncryl increased the stability of PCL in the blend, although the reduction occurred with a further increase in the chain extender. Similarly, an increase in the Tmax of PEG was observed in the blend upon the addition of Joncryl. Thus, it could be inferred that Joncryl had a greater effect on the minor components of the blend systems. Chain extenders are known to increase the molecular weight of polymers during chain extension processes, which could possibly lead to polymers prolonging their melting temperature, thus improving the thermal stability of PCL and PEG in the blend. It can be seen that Joncryl tends to provide a compatibilization effect leading to gaining thermal stability [43].

### 3.5. Thermomechanical Properties

**Figure 7:**
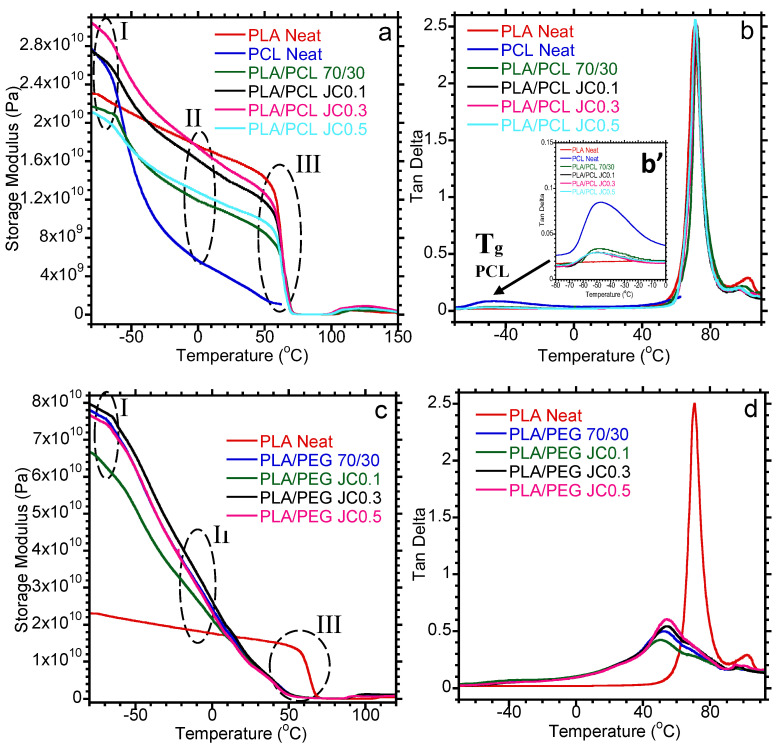
DMA properties storage modulus (**a**,**c**), tan delta (**b**,**d**) curves of PLA-based blends with varying content of Joncryl.

The thermomechanical behavior of the PLA-based blends containing Joncryl is shown in Figure 7. The application of PLA in various fields is restricted by its high stiffness and brittleness. Typically, ductile polymers, such as PEG and PCL, are added to increase the toughness of PLA. DMA is a vital tool to elucidate changes in the stiffness of PLA at various temperatures upon inclusion of ductile PEG and PCL. Figure 7a,c depicts the storage modulus as a function of temperature for PLA/PEG and PLA/PCL-based blends. Changes in the storage modulus are discussed at extremely low temperatures (−80 °C) (Region I), intermediate between the Tg of PLA and PEG and/or PCL (Region II), and at the Tg of PLA (~60 °C) (Region III). It is noteworthy that neat PEG could not run due to instrument limitations; therefore, the analysis of PEG was discussed based on the PLA/PEG blends. In contrast, neat PCL could run but not beyond 60 °C due to instrument limitation preventing the sample to break or melt. At extremely low temperatures (−80 °C), the mobility of the polymer chains is restricted, and the molecules have low internal energy. In this region, PCL showed a higher storage modulus than PLA, which could be attributed to the reinforcing effect of PCL crystallites in increasing the stiffness of PCL. However, with increasing temperature, the storage modulus of PCL plummets, and it is lower compared to PLA (Regions II and III). In Figure 7a, it is apparent that the storage modulus of PLA decreased with the inclusion of PCL at all temperatures, insinuating a reduction in the stiffness of PLA. Ferri et al. [16] also observed a decrease in approximately 19.3% in the storage modulus of the PLA/PCL blend containing 30 wt.% PCL at a temperature between 60 and 75 °C. The ductile PCL provides a plasticizing effect to PLA, allowing chain mobility within the blend. With the addition of Joncryl at 0.1 and 0.3 wt.%, the storage modulus of PLA/PCL blends increased relative to the neat PLA/PCL blend. The investigated storage modulus of the blends does not show any trend over a whole temperature range of the analysis. Therefore, no proper conclusions can be drawn about the influence of blending and the addition of Joncryl on the storage modulus. Similar results were also observed by Mofokeng et al. [44].

However, beyond regions II and III (PLA Tg), the storage modulus of PLA is higher compared to the respective blends. A higher concentration of Joncryl (0.5 wt.%) showed a similar behavior as the neat PLA/PCL blend. The tan delta curves were used to evaluate changes in the Tg of PLA and PCL. In Figure 7b, it is clear that the Tg of PLA did not change with the addition of PCL and Joncryl, suggesting a lower plasticization of PCL by increasing the mobility of the PLA chains. However, the effects of PEG on the thermomechanical properties of PLA were investigated (Figure 7c). PLA/PEG blends showed higher storage modulus compared to PCL-based blends in regions I and II. In region I, the neat PLA/PEG blend showed an approximately 4-fold increase in storage modulus with respect to PLA. The dramatic increase in the storage modulus of PLA/PEG blends could be attributed to the high crystallinity of PEG. PEG has a crystallinity of ~ 86%, which explains the high storage modulus at lower temperatures.

Moreover, both PEG and PCL show their melting temperatures closer to the Tg of PLA; thus, the storage modulus of the blends at region III is lower when compared to PLA because both ductile polymers exhibit high chain mobility. As expected, blending PLA with PEG decreases the storage modulus of PLA around 30–45 °C, attributed to the plasticization effect of PEG [5]. The addition of Joncryl at 0.1 wt.% showed a reduction in the storage modulus of the blend (especially in region I), whereas with the addition of 0.3 and 0.5 wt.%, there was no significant change with respect to the neat PLA/PEG blend. The plasticization effect of PEG could be noticed from the tan delta curves in Figure 7d. A shift towards lower temperatures in Tg of PLA could be observed, implying an increase in the mobility of PLA chains. The addition of Joncryl, however, did not show a significant influence on the Tg of PLA in the blend. Clearly, PEG has a higher plasticization effect than PCL.

### 3.6. Mechanical Properties

**Figure 8:**
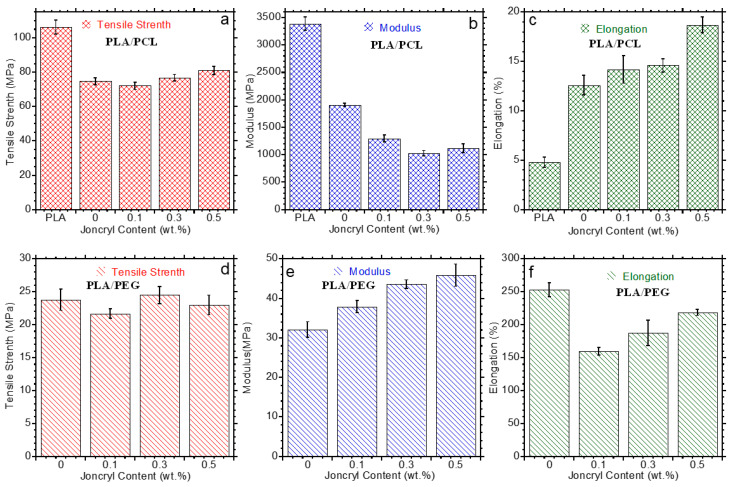
Mechanical properties tensile strength (**a**,**d**), modulus (**b**,**e**), and elongation (**c**,**f**) of PLA-based blends with varying content of Joncryl.

Although PLA has the potential to replace many conventional polymers in various applications, its inherent brittleness and stiffness remain major challenges, which impede its widespread utilization for numerous purposes. Blending with ductile polymers is considered a feasible strategy to reduce the brittleness of PLA, albeit this depends solely on the interfacial adhesion between PLA and the ductile matrix. Herein, the effects of PEG and PCL inclusion on the stiffness, strength, and toughness of PLA were investigated. The values of the mechanical properties of PLA/PCL and PLA/PEG based blends are summarized in Table 5 and Table 6, respectively. The tensile strength of the prepared samples is shown in Figure 8a,d. It is observed that the tensile strength of PLA decreased with the addition of PCL and PEG. The addition of Joncryl in both blend systems did not show any significant effect with respect to the neat blends.

Changes in tensile modulus provide an indication of the stiffness of a material. Figure 8b,e depicts the tensile modulus of PLA/PCL and PLA/PEG blends containing Joncryl. In this case, PLA is rigid, stiff, and has a high tensile modulus of 3390.2 MPa. The tensile modulus decreased to 1908.2 and 32 MPa upon the addition of PCL and PEG, respectively. This is facilitated by the plasticization effect of ductile PCL and PEG, which improves the drawability of PLA/PCL and PLA/PEG blends during tensile deformation. However, with the addition of Joncryl in both blend systems, an opposite trend could be observed (Figure 8b,e). The tensile modulus of PLA/PCL blends showed a decreasing trend, while the PEG-based blend showed an increasing trend with increasing Joncryl content. In the PLA/PCL system, although Joncryl induces interfacial adhesion, the toughening effect of PCL preponderates the chain stiffening caused by Joncryl in forming crosslinks, side chains, and branches, while in the PLA/PEG blend, the chain stiffening caused by the Joncryl effect predominates. Hence, the increase in the tensile modulus of the PLA/PEG blend system.

The changes in the toughness of PLA are depicted in Figure 8c,f, which show the plots of the tensile strain against the Joncryl content. PLA is brittle and stiff; hence, it showed tensile strain below 5%. However, it could be observed that the toughness of PLA/PCL and PLA/PEG was higher compared to that of neat PLA. With the incorporation of PCL, the tensile strain increased from 5% to 14.1%, as shown in Figure 8c. On the other hand, the inclusion of PEG showed a super-toughening effect, increasing the tensile strain from 5% to 250%. This is attributed to the increased chain mobility of PLA by PEG, which led to an increased macromolecular chain extension upon loading under tensile testing. These observations are in accordance with the observed changes in the tensile modulus of the neat blends. Ferri et al. [16] observed an increase in tensile strain from 8% for PLA up to 70% for PLA/PCL blend with 20–30 wt.% PCL. The improvement in ductile properties brought about by inclusion of PCL is good without compromising the mechanical resistance properties of immiscible blends. The addition of PEG as a plasticizer to PLA was reported to be successful in increasing the tensile strain by Mohapatra et al. [14], in which they observed that the presence of PEG reduces the brittle nature of PLA. Generally, the tensile strain of the blend system depends on the compatibility of the blend matrices. In this case, Joncryl was added to improve the compatibility of PLA with PEG and PCL. Both blends showed similar behavior when Joncryl was added. The tensile strain showed an increasing trend with the inclusion of Joncryl in PLA/PCL and PLA/PEG blends. In both blends, a significant increase is observed at 0.5 wt.% of Joncryl. In PLA/PCL blends, the tensile strain increased from 14.1% to 19%, while in PEG-based blends, the strain increased from 160% to 200%. In the case of PLA/PCL, a dramatic decrease in PCL droplets was observed and was attributed to the compatibility between blend matrices. In addition, the crystallinity was destroyed at 0.5 wt.% of Joncryl. This observation is attributed to the improved interfacial adhesion between PLA and PCL when Joncryl was added. The improvement in compatibility and interfacial adhesion between PLA and PCL means that the transfer of stress and strain from PLA matrix to PCL domains was more efficient after the addition of the chain extenders [33]. In the PLA/PEG blend, an initial decrease in toughness was noticed at 0.1 wt.% of Joncryl. The decrease observed is attributed to the formation of long branches, side chains, and lightly crosslinked structures, which restrict the elongation of the blend system [30]. However, with further addition of Joncryl content, the tensile strain increased, and this could be associated with decreased crystallinity of the blend, as observed from the XRD analysis. The mechanical properties of polymer blends depend on various factors, including interfacial adhesion and crystallinity. Therefore, there is a competition of these factors in determining the load-bearing behavior of the blend systems. Overall, it can be inferred that 0.5% Joncryl efficiently induced compatibility between PLA and PCL and/or PEG.

## 4. Conclusions

This study investigated the properties of PLA-based blends containing Joncryl as a compatibilizer. It was shown that the addition of Joncryl to both systems influenced the morphological and crystallinity as well as the thermal and mechanical performance of the blends. The morphological studies of the PLA/PCL blend revealed that the droplet size of PCL decreases with the addition of Joncryl. In the PLA/PEG blend system, a co-continuous morphology was observed with the addition of 0.1 and 0.3 wt.% Joncryl. The crystallinity of PCL and PEG in both blend systems decreased with the addition of Joncryl, while the thermal stabilities showed their dependence on the addition of Joncryl. It was further noticed that 0.5 wt.% Joncryl was more effective in improving compatibility, hence the higher toughness was observed for PLA/PCL and PLA/PEG blends compared to that of PLA. The stiffness of the PLA/PCL blend decreased with the inclusion of Joncryl, while the stiffness of the PLA/PEG blend increased with increasing Joncryl content. Overall, the addition of Joncryl in PLA-based blends showed a promising approach to improving the properties of PLA blended with ductile polymers such as PCL and PEG.

## Figures and Tables

**Table 1:** Sample abbreviations and compositions.

Sample Name	Composition	Blend Ratio (PLA:PCL)	Blend Ratio (PLA:PEG)	Joncryl (wt.%)
PLA Neat	PLA	100:0	100:0	-
PCL Neat	PCL	0:100	-	-
PEG Neat	PEG	-	0:100	-
PLA/PCL 70/30	PLA/PCL	70:30	-	-
PLA/PCL JC0.1	PLA/PCL/Joncryl	70:30	-	0.1
PLA/PCL JC0.3	PLA/PCL/Joncryl	70:30	-	0.3
PLA/PCL JC0.5	PLA/PCL/Joncryl	70:30	-	0.5
PLA/PEG 70/30	PLA/PEG	-	70:30	-
PLA/PEG JC0.1	PLA/PEG/Joncryl	-	70:30	0.1
PLA/PEG JC0.3	PLA/PEG/Joncryl	-	70:30	0.3
PLA/PEG JC0.5	PLA/PEG/Joncryl	-	70:30	0.5

**Table 2:** DSC data of PLA/PCL-based blends obtained from cooling and second heating curves.

Sample Name	Tm PLA (°C)	ΔH_m_ PLA (J/g)	Tcc PLA (°C)	Tm PCL (°C)	ΔH_m_ PCL (J/g)	Tc PCL (°C)	X_c_ PLA (%)	X_c_ PCL (%)
PLA Neat	151.0 ± 0.1	20.4 ± 0.5	125.9 ± 0.2	-	-	-	21.8 ± 0.5	-
PCL Neat	-	-	-	57.8 ± 0.3	62.7 ± 0.7	30.1 ± 0.3	-	46.1 ± 0.5
PLA/PCL 70/30	148.9 ± 0.6	17.3 ± 0.6	119.1 ± 1.0	57.0 ± 0.4	16.9 ± 0.2	29.8 ± 0.5	26.4 ± 1.0	41.4 ± 0.5
PLA/PCL JC0.1	149.1 ± 0.1	20.7 ± 0.9	117.0 ± 1.1	57.3 ± 0.0	14.8 ± 1.2	29.3 ± 0.8	31.6 ± 1.3	36.3 ± 3.0
PLA/PCL JC0.3	149.7 ± 0.2	21.1 ± 2.5	118.1 ± 0.3	57.3 ± 0.2	15.7 ± 1.5	28.2 ± 0.0	32.2 ± 3.9	38.5 ± 3.7
PLA/PCL JC0.5	149.6 ± 0.4	19.6 ± 0.7	118.4 ± 1.5	57.2 ± 0.1	12.8 ± 0.1	-	29.9 ± 1.1	31.4 ± 0.3

The experiment of neat PLA, neat PCL, and their blends was conducted twice for each sample.

**Table 3:** DSC data of PLA/PEG-based blends obtained from cooling and second heating curves.

Sample Name	Tm PLA (°C)	ΔH_m_ PLA (J/g)	Tcc PLA (°C)	Tm PEG (°C)	ΔH_m_ PEG (J/g)	Tc PEG (°C)	X_c_ PLA (%)	X_c_ PEG (%)
PLA Neat	151.0 ± 0.1	20.4 ± 0.2	125.9 ± 0.5	-	-	-	21.8 ± 0.5	-
PEG Neat	-	-	-	66.1 ± 0.4	170.3 ± 1.1	44.1 ± 0.5	-	86.4 ± 0.5
PLA/PEG 70/30	153.7 ± 1.5	27.8 ± 0.3	-	-	-	36.0 ± 1.2	42.4 ± 0.4	-
PLA/PEG JC0.1	153.0 ± 0.6	24.9 ± 0.6	88.9 ± 0.3	-	-	32.7 ± 1.0	38.0 ± 0.9	-
PLA/PEG JC0.3	152.3 ± 0.3	24.8 ± 0.4	88.2 ± 0.1	-	-	31.3 ± 1.0	37.8 ± 0.6	-
PLA/PEG JC0.5	151.6 ± 0.1	24.0 ± 0.2	88.2 ± 0.0	-	-	32.1 ± 0.5	36.6 ± 0.3	

The experiment of neat PLA, neat PEG, and their blends was conducted twice for each sample.

**Table 4:** Thermal parameters of PLA-based blends with various content of Joncryl.

Sample Name	Tmax PLA (°C)	Tmax PCL (°C)	Tmax PEG (°C)
PLA Neat	362.2 ± 1.9	-	-
PCL Neat	-	402.1 ± 4.8	
PEG Neat	-		390.2 ± 2.3
PLA/PCL 70/30	361.3 ± 5.1	377.4 ± 4.3	-
PLA/PCL JC0.1	366.3 ± 3.6	401.6 ± 3.0	-
PLA/PCL JC0.3	365.1 ± 0.5	390.0 ± 0.7	-
PLA/PCL JC0.5	364.0 ± 1.7	384.5 ± 0	-
PLA/PEG 70/30	355.9 ± 2.6	-	304.9 ± 9.9
PLA/PEG JC0.1	352.4 ± 3.1	-	385.4 ± 11.3
PLA/PEG JC0.3	360.0 ± 9.1	-	377.9 ± 3.6
PLA/PEG JC0.5	358.6 ± 6.6	-	379.4 ± 2.9

(a) The experiment of neat PLA, neat PCL, and their blends was conducted twice for each sample. (b) The experiment of neat PLA, neat PEG, and their blends was conducted twice for each sample.

**Table 5:** Mechanical properties of PLA/PCL-based blends.

Sample Name	Tensile (MPa)	Modulus (MPa)	Elongation (%)
PLA Neat	106.3 ± 4.1	3390.2 ± 121.7	4.8 ± 0.5
PCL Neat	37 ± 1.9	64.7 ± 4.2	660.5 ± 54.1
PLA/PCL 70/30	74.8 ± 2.0	1908.2 ± 33.5	12.6 ± 1.0
PLA/PCL JC0.1	72.1 ± 2.1	1295.7 ± 65.9	14.2 ± 1.4
PLA/PCL JC0.3	76 ± 1.9	1030.8 ± 47.0	14.6 ± 0.7
PLA/PCL JC0.5	81.1 ± 2.4	1120.1 ± 80.2	18.7 ± 0.8

**Table 6:** Mechanical properties of PLA/PEG-based blends.

Sample	Tensile (MPa)	Modulus (MPa)	Elongation (%)
PLA Neat	106.3 ± 4.1	3390.2 ± 121.7	4.8 ± 0.5
PLA/PEG 70/30	23.8 ± 1.6	32.1 ± 2.0	253.2 ± 10.8
PLA/PEG JC0.1	21.7 ± 0.7	37.9 ± 1.5	160 ± 5.9
PLA/PEG JC0.3	24.5 ± 1.3	43.6 ± 1.1	187.6 ± 19.5
PLA/PEG JC0.5	23 ± 1.5	45.9 ± 2.8	218.7 ± 4.6

## Data Availability

Not applicable.

## Supplementary Materials

The following supporting information can be downloaded at: https://www.mdpi.com/article/10.3390/polym15092230/s1, Figure S1: SEM images of the cryogenically fractured surface morphologies of (a) PLA/PCL 80/20, (b) PLA/PCL 70/30, and (c) PLA/PCL 60/40.; Figure S2: Mechanical properties of PLA/PCL blends: (a) tensile strength, (b) modulus, and (c) elongation.
