# Supplementary material for: Investigation of the Effects of Chain Extender on Material Properties of PLA/PCL and PLA/PEG Blends: Comparative Study between Polycaprolactone and Polyethylene Glycol
Source: Polymers (Basel). 2023 May 8;15(9):2230. doi: 10.3390/polym15092230 (PMC10181129; doi:10.3390/polym15092230)
Supplement: Supplementary file 1 [file polymers-15-02230-s001.zip › polymers-2259395-supplementary.pdf]

polymers

Supplementary Materials

Investigation of the Effects of Chain Extender on Material Properties of PLA/PCL and PLA/PEG Blends: Comparative Study between Polycaprolactone and Polyethylene Glycol

Karabo Innocent Matumba [1,2], Mpho Phillip Motloung [1,3], Vincent Ojijo [1], Suprakas Sinha Ray [1,3,*] and Emmanuel Rotimi Sadiku [2]

1 Centre for Nanostructures and Advanced Materials, DSI-CSIR Nanotechnology Innovation Centre, Council for Scientific and Industrial Research, Pretoria 0001, South Africa
2 Institute of NanoEngineering Research, Department of Chemical, Metallurgical and Materials Engineering, Polymer Division, Tshwane University of Technology, Pretoria 0001, South Africa
3 Department of Chemical Sciences, University of Johannesburg, Johannesburg 2028, South Africa
* Correspondence: rsuprakas@csir.co.za

1. Phase Morphology

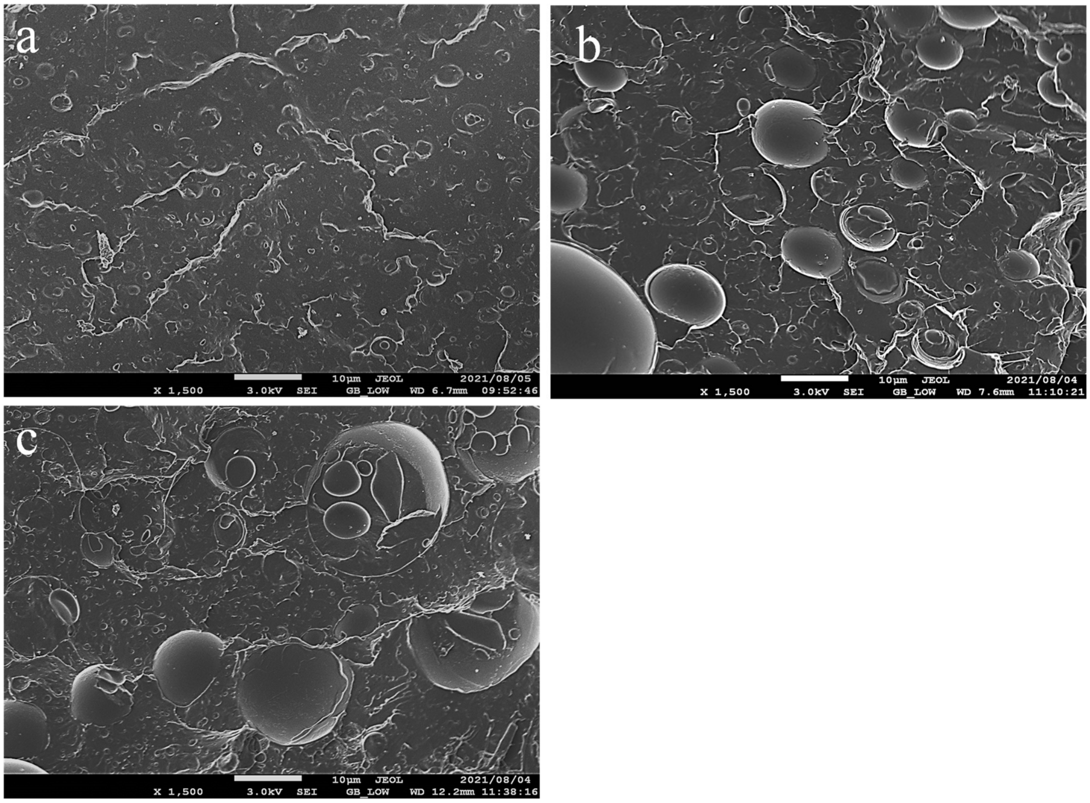

Figure S1. SEM images of the cryogenically fractured surface morphologies of (a) PLA/PCL 80/20, (b) PLA/PCL 70/30, and (c) PLA/PCL 60/40.

Figure S1 shows the cryogenically fractured surface morphologies of PLA/PCL blends with varying PCL content at 20, 30, and 40 wt.%. This figure shows a matrix-dispersed morphology, where PCL droplets are dispersed in a continuous PLA matrix. In

Figure S1(a), with the addition of 20 wt.% PCL, the image shows that small PCL droplets are heterogeneously distributed within the PLA matrix. However, when the PCL content is increased to 30 wt.%, the coalescence of PCL droplets is observed within the PLA matrix as shown in **Figure S1(b)**. Therefore, it shows a better distribution of PCL droplets dispersed within the continuous PLA matrix. In **Figure S1(c)**, the size of PCL droplets increased with 40 wt.% of PCL, showing a large droplet size. Overall, PLA/PCL 70/30 wt.% shows a good morphology with better distribution of PCL droplets. The matrix-dispersed morphology increases the toughness of PLA as the primary polymer within the blend.

2. Mechanical Properties

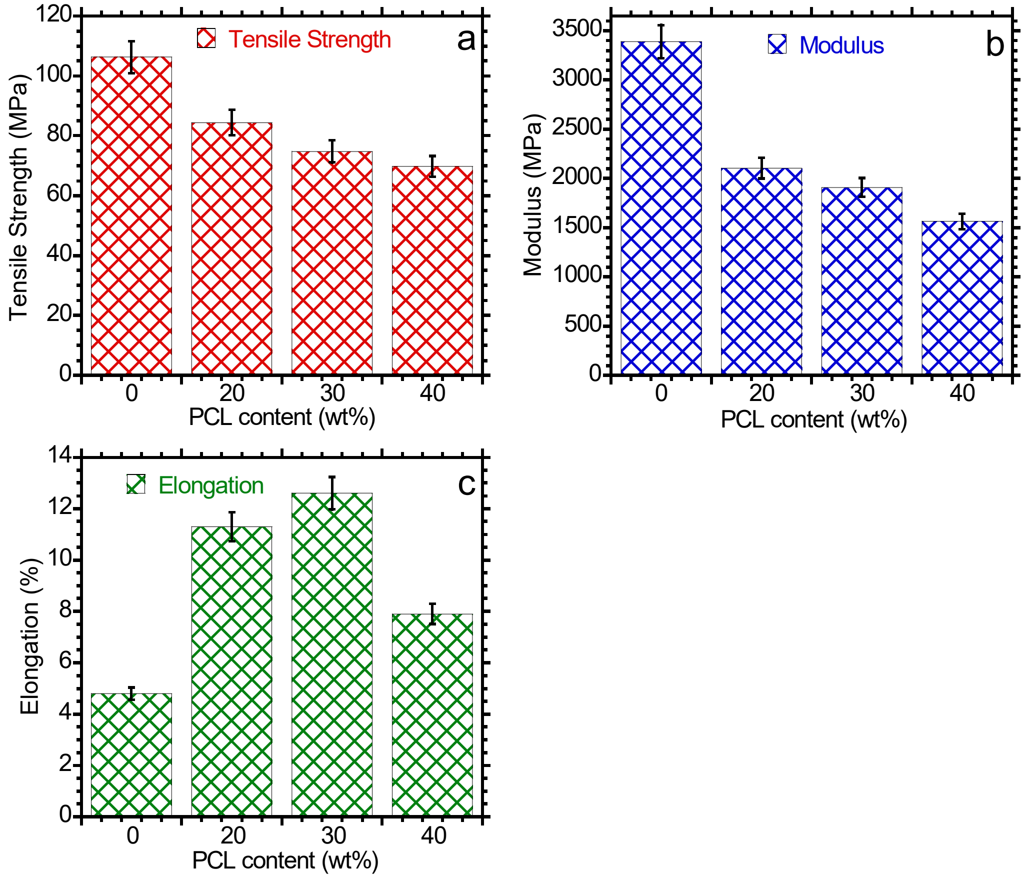

Figure S2. Mechanical properties of PLA/PCL blends: (a) tensile strength, (b) modulus, and (c) elongation.

PLA has a good potential to replace conventional polymers. However, it still suffers from high brittleness. Therefore, blending it with ductile polymers is reported to be a promising method to reduce the brittleness. The mechanical properties of the PLA/PCL blends studied are shown in **Figure S2**. The tensile strength of the PLA/PCL blends is shown in **Figure S2(a)**. It is observed that the tensile strength of PLA decreases with increasing PCL content. **Figure S2 (b) shows** the tensile modulus of PLA/PCL blends. This provides information about the stiffness of the material. The stiffness of the material decreased with increasing PCL content. In **Figure S2(c)**, the elongation-at-break is shown, indicating the toughness behaviour of the material. The elongation-at-break increased with the addition of 20 and 30 wt.%, showing an increase in the toughness of the material. However, when 40 wt. PCL was added to PLA, the toughness decreased compared to when 20 and 30 wt.% PCL were added. In summary, the addition of PCL to the PLA matrix

increases the elongation at break of the PLA, indicating a greater toughness of the material with 30 wt.% proving to be enough to improve the toughness of the PLA.

3. Conclusions

The study investigated PLA/PCL blends containing 20, 30, and 40 wt.% PCL content. The results showed that the PCL content influenced the final morphological behaviour of the material, with 30 wt.% of PCL showing a better distribution of PCL droplets. The mechanical properties of the blends show that 30 wt.% PCL is enough to increase the toughness of the material. These results proved to be enough to give knowledge in choosing the optimum blend.
